# THERAPEUTIC APPROACH AND ITS RELATIONSHIP WITH SOCIAL AND ECONOMIC CHARACTERISTICS OF USERS OF CENTERS FOR PSYCHOSOCIAL CARE FOR CHILDREN AND ADOLESCENTS

**DOI:** 10.1590/1984-0462/;2017;35;4;00007

**Published:** 2017

**Authors:** Ana Paula Soares Gondim, Ana Paula Pessoa Maciel, Mirian Parente Monteiro

**Affiliations:** a Universidade Federal do Ceará, Fortaleza, CE, Brasil.

**Keywords:** Mental health, Child, Therapeutic, Saúde Mental, Criança, Terapêutica

## Abstract

**Objective:**

To analyze the therapeutic approach and its relationship with the economic and social characteristics and the care of children in Centers for Psychosocial Attention.

**Methods:**

Descriptive study with a sample of 294 children monitored in two Centers for Psychosocial Attention to Children and Adolescents in Fortaleza, Ceará, Northeast Brazil. The study was conducted from February to December, 2012. Participants were accompanied by their parents or caregivers. Data were collected in a structured questionnaire containing social, economic and care variables. The bivariate analysis used the χ^2^ test to test the association between variables.

**Results:**

In this study, 292 children aged 3-12 were selected, following the order of attendance at the service, most of them male (74.3%) and belonging to social classes D and E (89.3%). The most frequent diagnosis referred to by the caregivers was mental disorders. Three different therapeutic approaches were identified: pharmacological approach (44.5%); non-pharmacological approach (11.6%); association of both techniques (43.8%). For all therapeutic approaches, there was association with the variable living situation (p=0.021), as well as with the variables, “improving” with the treatment (p=0.002) and “problems” with the treatment (p=0,004).

**Conclusions:**

It was possible to highlight that the associated therapeutic approach (pharmacological and non-pharmacological treatment) provides more benefits to children. Therefore, associating the medicines to the psychotherapeutic practices may be recommended as a strategy in the mental health policy directed to children and adolescents.

## RESUMO

**Objetivo:**

Descrever a abordagem terapêutica e estabelecer a relação entre características sociais, econômicas e o cuidado de crianças nos Centros de Atenção Psicossocial Infantojuvenis.

**Métodos:**

Estudo descritivo com cálculo amostral de 294 crianças em acompanhamento nos dois Centros de Atenção Psicossocial Infantojuvenis em Fortaleza, no Ceará, no período de fevereiro a dezembro de 2012. Os participantes estavam acompanhados por seus cuidadores, pais ou responsáveis. Os dados foram coletados mediante aplicação de formulário validado para os cuidadores, contendo as variáveis sociais, econômicas e cuidado. A análise bivariada utilizou o teste χ^2^ para verificar diferenças entre as proporções, com nível de significância 5%, intervalo de confiança 95%.

**Resultados:**

Foram selecionadas 292 crianças de 3 a 12 anos, por ordem de atendimento no serviço, a maioria do sexo masculino (74,3%) pertencentes às classes D e E (89,3%). A hipótese diagnóstica mais frequente referida pelo cuidador foi a de transtornos mentais. Três formas de abordagens terapêuticas foram identificadas: abordagem medicamentosa (44,5%), abordagem não medicamentosa (11,6%) e combinação da abordagem medicamentosa com as técnicas psicoterapêuticas (43,8%). Houve significância estatística entre a presença de abordagem terapêutica e a situação de moradia (p=0,021), bem como com as variáveis, “melhora” com o tratamento (p=0,002) e “problemas” com o tratamento (p=0,004).

**Conclusões:**

O estudo permitiu evidenciar que a abordagem terapêutica combinada (tratamento medicamentoso e não medicamentoso) proporciona maior efeito benéfico às crianças. Desse modo, associar o medicamento às técnicas psicoterapêuticas pode constituir-se como uma das principais estratégias terapêuticas da política de saúde mental infantojuvenil.

## INTRODUCTION

Mental disorders are estimated in 12 to 29% of the children,^1^ ^,^ ^2^ among which there are: intellectual development and communication disorders; autism spectrum; attention/hyperactivity deficit; specific learning, motor, conduct, impulse control and disruption deficit; schizophrenia and other psychotic disorders, besides anxiety.^2^ ^,^ ^3^

The therapeutic approaches of disorders in childhood must be based on a psychosocial care clinic as a “broadened”, territorialized, inter and transdisciplinary clinic, addressed to the individual and his or her social and cultural context.^4^ These approaches should be constituted of multi and interdisciplinary activities, with the participation of educators, health professionals and family members, performing the individualized drug treatment and the non-drug and collective treatment.^5^

Drug treatments are based on the use of psychoactive drugs, according to the clinical consensus and empirical evidence adapted to the best practices and clinical experiences.^6^ Considering the non-drug treatments, the focus lies on socioeducational interventions, consisting of therapeutic groups, family guidance and family therapy.^5^ These interventions are a way to work with the humanization of the treatment, developing autonomy, co-responsibility, protagonism among the parties involved, solidarity with the connections established, respect to the rights of the users and the group participation in the management of the treatment.^7^ In some cases, it is necessary to adopt an immediate drug treatment, even though psychotherapeutic techniques are recommended as the first choice for the treatment of mental disorders in childhood.^8^

The Centers of Psychosocial Care for Children and Adolescents (CAPS i) integrate the mental health care network in the Unified Health System (SUS), with the objective of providing care to children who suffer from severe and persistent mental disorders, through an intensive treatment regime (daily), semi-intensive regime (frequent) or non-intensive regime (punctual).^9^ The objectives of the actions in these services should be planned and established based on the unique therapeutic project, and executed by a multiprofessional team, addressed to the recovery of the user, from admission to discharge, preventing medicalization.^9^ ^,^ ^10^ The multidisciplinary technical team is composed of psychologist, social worker, nurse, pharmacist, nursing assistant, clinical physician and psychiatrist, who provide care to the user and family members.^10^

The objective of this study was to describe the therapeutic approach and to establish the relationship between the social and economic characteristics and the care of children in CAPS i.

## METHOD

Descriptive study, with a quantitative approach, of a simple random sample representative of the children being followed-up in the two CAPS i in Fortaleza, Ceará, conducted from February to December, 2012.

The sample was measured using the calculation for finite populations in cross-sectional studies. A finite population of 2,883 patients was established, aged between 3 and 12 years old, registered in CAPS i until 2012. The age group was based on the Child and Adolescent Statute, which classifies as children those up to the age of 12.^11^ Considering a percentage of mental disorders (p=50.0%) for this population, the significance level fixed at 97.0% and the relative sampling error of 6.0%, the sample calculated was of 294 children.^12^ The selection of this sample took place sequentially in both services, on the days of care, as long as the participants were accompanied by their parents or tutors. The exclusion criteria were: participants who were not being followed-up at CAPS i, patients outside the age group established and those who were not accompanied by their parents or tutors. Two children were excluded for not being accompanied by their parents or tutors.

For data collection, a codified form was elaborated and previously tested in a pilot study, calibrated to obtain information about caretakers and patients. This form was applied to caretakers, parents or tutors who were with the child at the moment when they were waiting to be assisted.

The variables analyzed in the study as to the social and clinic characteristics of the children were: child’s age (in years), sex (male/female), social benefit (yes/no), and diagnostic hypothesis referred by the caretaker (mental disorders/behavioral disorders/emotional disorders/disorders that do not match the diagnosis/other conditions). The social and economic characteristics of caretakers and family were: caretaker’s age (in years), degree of kinship (father, mother, others), schooling (Elementary School/High School/ Higher Education), family economic class (B/C/D/E), family income (lower than or equal to R$ 622.00, or higher than R$ 622.00), type of family household (house/others), number of residents (1-4/5-12), and situation of the family household (property/others). The characteristics of the therapeutic approaches used in CAPS i were: drug treatment, non-drug treatment and combined treatment, referred by the caretaker. The characteristic about the knowledge of the treatment: improvement with treatment, problem with treatment, and family support to the treatment.

The variable social benefit was defined by the follow-up of users who receive a social benefit from the government (Bolsa-Família, Social Welfare and Passe Livre - Brazilian governmental programs) to CAPS i. The diagnostic hypothesis was classified based on the description of symptoms reported by the caretakers. The variable degree of kinship was defined according to the Brazilian Civil Code.^13^ The economic class variable followed the Brazilian Economic Classification Criterion, from the Brazilian Association of Research Companies.^14^ The family income amount was based on the minimum wage of 2012, worth of R$ 622.^15^ The variable number of residents was divided in two groups based on the mean number of inhabitants. The variable referring to the problem with the treatment aimed at identifying if the users presented any adverse effect related to the treatment. The variable family support to the treatment was described to identify if the family supported the therapeutic approach established for the user in CAPS i.

The data were stored with the statistical software EPI INFO™ *for Windows,* version 3.5.4 (Centers for Disease Control and Prevention*,* Atlanta, USA), and analyzed with the software STATA,, version 11 (Statacorp, Texas, USA). The data analysis plan began with a simple statistical analysis, presenting the proportions for the categorical variables and the measures of tendency (means) and dispersion (standard deviation) for the numerical variables. The bivariate analysis was conducted to compare the different therapeutic approaches (dependent variable) for the treatment of children followed-up in CAPS i in relation to the independent variables represented by all groups of variables. Therefore, the χ^2^ test was used, considering a 5% significance level and a 95% confidence interval. 

The study was approved by the Research Ethics Committee of Universidade de Fortaleza and approved under Protocol n. 189/2011.

## RESULTS

Among the 292 children selected for the study, most were male (74.3%), aged between 7 to 9 years old (45.5%) - mean age 8.1±0.5 years - and 63.5% had social benefits. As to caretakers, the main degree of kinship was mother (81.0%), aged from 22 to 39 years (56.2%), who completed Elementary School (62.3%). Most families belonged to economic classes D and E (89.3%), with mean family income of R$ 756.40, ranging from R$ 99 to R$ 5,000. Almost all families lived in a house (94.1%), of their own (61.03%), with an average of 4.4 residents per household (Table 1).

**Table 1:** Distribution of children and caretakers according to social and economic characteristics in the Centers of Psychosocial Care for Children and Adolescents.

	n	%	Mean	Standard deviation
Sex (n=292)				
Male	217	74.3		
Age (in years) (n=292)				
3-6	71	24.3	8,1	0.5
7-9	133	45.5		
10-12	88	30.2		
Social benefit (n=290)				
Sim	184	63.5		
Age of the caretaker (years) (n=290)				
22-39	163	56.2	39,1	0.5
40-78	127	43.8		
Degree of kinship of the caretaker (n=290)				
Mother	235	81.1		
Father	21	7.2		
Others	34	11.7		
Caretaker’s schooling (n=289)*				
Elementary School	180	62.3		
High/Higher Education	109	37.7		
Economic classification (n=290)				
B + C	31	10.7		
D + E	259	89.3		
Family income (R$) (n=277)**				
Lower than or equal to R$ 622.00	162	58.5	756.40	33.00
Lower than R$ 622.00	115	41.5		
Type of household (n=290)				
House	273	94.1		
Number of residents (n=290)				
1-4	256	88.3	4.4	0.1
5-12	34	11.7		
Housing situation (n=290)				
Own house	177	61.1		
Others	113	38.9		

*7 informants were illiterate. **In 2012, the minimum wage was R$ 622.00.

The diagnostic hypotheses most frequently referred by the caretaker were mental disorders (44.9%), and the most used therapeutic approach was the drug treatment (44.5%), followed by the combination of drug and non-drug treatment (43.8%) and non-drug treatment (11.6%). In the study, most people improved with the drug treatment (80.6%), had no problems with the treatment (75.0%) and the family supported the treatment (96.7%) (Table 2).

**Table 2:** Distribution of children according to the characteristics of the diagnostic hypothesis reported by the caretakers, therapeutic approach, improvement with treatment, problem with treatment and family support to treatment, identified by the caretakers in the Centers of Psychosocial Care for Children and Adolescents.

	n	%
Diagnostic hypothesis referred by caretakers (n=363)*		
Mental disorders	163	44.9
Behavioral disorders and emotional disorders	88	24.2
Disorders not in accordance with diagnosis	38	10.5
Others	74	20.4
Therapeutic approach (n=292)		
Drug treatment	130	44.5
Non-drug treatment	34	11.6
Both treatments	128	43.8
Improvement with treatment (n=288)		
Yes	232	80.6
No	56	19.4
Problem with treatment (n=288)		
Yes	72	25.0
No	216	75.0
Family supporting treatment (n=282)		
Yes	267	96.7
No	15	5.3

*More than one hypothesis was mentioned by the caretaker.

Table 3 compares the different therapeutic approaches to the social and economic characteristics of the children, caretakers and families. The male sex was prevalent among the different approaches. The non-drug treatment was prevalent in the age group between 3 and 7 years, whereas the drug treatment and the combination of both prevailed in the age group of 8 to 12 years. The social benefit granted to the children was present in the three groups of treatment.

**Table 3:** Comparison of the therapeutic approach with the social, economic and care characteristics used in the Centers of Psychosocial Care for Children and Adolescents.

	Therapeutic approach								
	Drug treatment		Non-drug treatment		Combined treatment		Total		p-value
	n	%	n	%	n	%	n	%	
Sex (n=292)									
Male	91	70	27	79.4	99	77.3	217	74.3	0.107
Female	39	30	7	20.6	29	22.7	75	25.7	
Age (years) (n=292)									
3-7	47	36.2	19	55.8	54	42.2	120	41.1	0.107
8-12	83	63.8	15	42.2	74	57.8	172	58.9	
Social benefit (n= 292)									
No	40	31.1	15	44.1	51	40.2	106	36.6	0.189
Yes	89	68.9	19	55.9	76	59.8	184	63.4	
Caretaker’s age (years) (n=290)									
22-39	75	58.1	18	52.9	70	55.1	163	56.2	0.827
40-78	54	41.9	16	47.1	57	44.9	127	43.8	
Caretaker’s schooling (n=289)									
Elementary school	79	61.7	20	58.8	81	63.8	180	62,3	0.845
High School/Higher Education	49	38.3	14	41.2	46	36.2	109	37.7	
Economic classification (n=290)									
B + C	10	7.7	5	14.7	16	12.6	31	10.7	0.301
D + E	119	92.3	29	85.3	111	87.4	259	89.3	
Family income (n=277)									
≤ R$622,00	69	55.6	16	51.6	77	45.0	162	54.9	0.346
R$622,00	55	44.4	15	48.4	63	36.9	133	45.1	
Type of household (n=290)									
House	123	95.3	32	94.1	118	92.9	273	94.1	0.681
Others	6	4.7	2	5.9	9	7.1	17	5.9	
Number of residents (n=290)									
1-4	80	62.1	20	58.8	83	65.3	183	63.1	0.734
5-12	49	37.9	14	41.2	44	34.7	107	36.9	
Housing situation (n=290)									
Own house	74	57.4	28	82.3	75	59.1	177	61.0	0.021
Others	55	42.6	6	17.7	52	40.9	113	39.0	

Among caretakers, the age group of 22 to 39 years old and the Elementary school level were the clearest. As to the family characteristics, classes D e E were prevalent, as well as ­family income below R$ 622, living in their own households, with less than 4 residents per household. These characteristics were the most frequent ones, and were not related with any type of therapeutic approach. Only the variable “housing situation” presented statistical significance (p=0.021), when related with the therapeutic approach (Table 3).

Table 4 compares the different therapeutic approaches regarding the knowledge of the treatment, using the variables “improvement with the treatment”, “problem with the treatment”, and “family support to the treatment”, which characterizes such knowledge. It was observed that most children (83.9%) improved when the approach used for treatment was the combination of drug and non-drug treatments. One third of the children presented problems with the treatment, both drug or combined treatment, whereas 2.9% presented non-pharmacological problems with the treatment. The variables “improvement with the treatment” (p=0.002) and “problems with treatment” (p=0.004) presented statistical significance in relation to the therapeutic approach.

**Table 4:** Comparison of the therapeutic approach with the improvement and the onset of problems during the treatment adopted in the Centers of Psychosocial Care for Children and Adolescents.

	Therapeutic approach								
	Drug treatment		Non-drug treatment		Combination of treatments		Total		p-value
	n	%	n	%	n	%	n	%	
Improvement with treatment									
No	22	16.9	14	41.2	20	16.1	56	19.4	0.002
Yes	108	83.1	20	58.8	104	83.9	232	80.6	
Problem with treatment									
No	91	70.0	33	97.1	92	74.2	216	75.0	0.004
Yes	39	30.0	1	2.9	32	25.8	72	25.0	
Family supporting the treatment									
No	7	5.51	1	2.9	7	5.8	15	6.5	0.801
Yes	120	94.5	33	97.1	114	94.2	267	93.5	

## DISCUSSION

The study revealed that the therapeutic approaches in CAPS i of Fortaleza are focused on the treatment with psychoactive drugs, and most of the service is mainly medical, which demotes the hegemony of the biomedical model of SUS. Such a fact would be opposed to the principles of the psychiatric reform^16^ and the proposal of children and adolescent mental health policy, such as interdisciplinary, flexibility and versatility in patient care.^17^ The non-drug treatment encourages greater interaction of health professionals and users providing the development of healthy attitudes for all involved, as well as the search for greater autonomy on the part of users.^18^

From the caretakers’ point of view, it was possible to observe that the families support the treatment of children in CAPS i, regardless of the approach adopted. The families demonstrate little participation in the decision-making process of the treatment. When asked about improvement, problems and difficulties in the treatment, they do not express any type of complaint or disagreement, indicating a behavior of acceptance, regardless of the conduct adopted. However, the caretakers reported presenting problems with the treatment concerning the use of psychoactive drugs, which suggests adverse events. Even though this study registered problems such as diarrhea and somnolence, the causality of these events was not assessed.^19^

The social and economic characteristics of the children assisted in both CAPS i show that these come from economically disadvantaged social classes (classes D and E), with family income lower than one minimum wage, living in their own houses, with less than four residents per household, depending on social security and attending CAPS i, accompanied by their mothers, who are young, married and with low schooling. These characteristics seem to be determinant in the choice of type of treatment addressed to these children. Besides, it is important to mention the early age of this group (mean age of 8.1 years), and the fact that they are mostly male participants. The prevalence of the male gender also occurs in other studies conducted in CAPS i in the country: São Paulo (61.2%),^20^ Recife (83.3%)^21^ and in the Southeast region (62.8%).^22^ A study conducted in Psychology services also points to a higher number of boys (59.7%) using this type of service, as found in this study, even though it was conducted in CAPS i.^22^

The mean age of this study was 8.1 years, lower than the one found in another study conducted in a CAPS i in São Paulo, which was 9.4 years.^20^ The prevalent age group in the study was between 8 and 12 years, higher than that in another study conducted in CAPS i in Recife, Pernambuco, in which the age group was from 4 to 5 years.^21^ The authors state that the proportion of mental disorders in childhood tends to increase according to age, due to the difficulty of professionals who work with children, especially pediatricians, to define diagnoses.^23^

Most children accompanied in CAPS whose data were analyzed belong to families of social classes D and E with income below R$ 622.00 and receive social benefit. These results are also shown in other studies, such as a study in New Zealand that points out that the social features of the family influence, in a multidimensional way, the children’s mental disorders.^24^

The complementation of the family income associated with the benefit favors the conditions to look for specialized services such as CAPS i. The unfavorable financial conditions make it difficult to access the treatment and favor abandonment, which may lead to worse health status.^25^

In this study, mothers were younger, married, and with lower schooling. Most times, they accompany their children during treatment and to CAPS i care. At the end of the XVIII century until the beginning of the XIX century, Brazil experienced the hygienist movement, in which the female figure began to be seen as the main “protector of the health” of the children, and that persists until the current days.^5^ ^,^ ^26^ Studies try to explain the role of women in care of people with psychic suffering in the household environment, and relate a feminization of the burden of attending the person with mental disorder making it clear that the provision of care for family members is a matter of gender historically produced, which sees the women as excellent caretakers, both for sick and healthy family members.^27^

The complaints of mental disorders and behavioral changes in children, with the awareness of their caretakers, may be associated with cultural and environmental dimensions, requiring children and adolescents in current days to receive a more careful evaluation in schools for health services before being referred to mental health services.^25^ Therefore, it is up to the professional to assess the patient carefully, and to establish the limit that separates the matters inherent to development from those that configure, together, a mental disorder.

The main limitation identified in the conduction of this study was in the difficulty for the caretakers to distinguish what was asked about the problem in treatment and, consequently, what was considered as an improvement in treatment. In the beginning of the study, it was easy to notice the difficulties from the caretakers to distinguish the assistance provided to the child from what should be considered as a therapeutic benefit. In order to change this situation, there was an adjustment in the instrument used to interview the caretakers, using a more appropriate language for the group interviewed.

The study showed that the associated therapeutic approach (drug and non-drug treatments) provides more benefits to children (84%), and this situation is reported more often by the caretakers. Therefore, from the perspective of caretakers, combining the medicine to psychotherapy techniques can be one of the main therapeutic strategies in children and adolescent mental health policies.^9^ These findings offer subsidies for reflections regarding the therapeutic approaches used in CAPS i, which may subsidize strategies enabling better care to patients and families, promoting the development of autonomy, health and well-being.
